# Dental services access and oral health improvement in Gypsy, Romany and Traveller communities: a scoping review

**DOI:** 10.1038/s41405-026-00459-4

**Published:** 2026-07-25

**Authors:** John Tredinnick-Rowe, Joelle Booth, Lorna Burns, Janine Doughty, Afsha Musa, Robert Witton, Martha Paisi

**Affiliations:** 1 Peninsula Dental Social Enterprise, Plymouth, UK; 2 School of Nursing and Midwifery, University of Plymouth, Plymouth, UK; 3 Peninsula Dental School, University of Plymouth, Plymouth, UK; 4 School of Dentistry, University of Liverpool, Liverpool, UK; 5 Dental Public Health Training Programme, Southwest, UK

**Keywords:** Oral hygiene, Dental treatments

## Abstract

**Background:**

Gypsy, Romany, and Traveller (GRT) populations experience poor oral health, with a recognised unmet need for care compounded by multiple barriers to accessing dental services. This scoping review aims to identify and map the available evidence, as well as gaps in the literature, relating to oral health improvement and access to care among GRT populations in Europe.

**Methods:**

Studies published in English relating to oral health improvement and/or access to care among GRT populations were included. Electronic databases searched comprised Embase (Ovid), Scopus (Elsevier), Web of Science (Clarivate), and Dentistry and Oral Sciences Source (EBSCOhost). In addition, a grey literature search was conducted. Titles and abstracts were screened against the eligibility criteria, followed by full-text screening, by two independent reviewers. Included studies were analysed using thematic analysis, and findings were tabulated to illustrate study characteristics and outcomes related to oral health improvement and access to care.

**Results:**

Of the initial 436 search results, 18 publications met the inclusion criteria and were included in the review. Overall, there was a paucity of literature addressing oral health access and improvement among GRT populations, with even fewer evaluated programmes designed to address these challenges. Identified barrier-related themes included limited health literacy, structural barriers to accessing care, prohibitive costs, and population mobility, which affected continuity of care, routine engagement with services, and registration. Fewer facilitating factors were identified. Community health workers were most frequently reported as supporting access to services and promoting oral health within these populations. Interventions such as mobile dental units appeared effective but were resource-intensive. The most sustained benefits in GRT communities were associated with initiatives that fostered trust between communities, health services, and care providers.

**Conclusions:**

GRT communities experience significant disadvantage in accessing dental care, with substantial barriers to meeting their treatment needs. To address the limited evidence base for oral health programmes, further research, particularly focused on oral health improvement, should be conducted with these populations.

## Background

Gypsy, Roma and Traveller (GRT) is an umbrella term that is used to define those who self-identify as belonging to a group who traditionally have a culture of nomadism and face discrimination and social stigma [1]. The term GRT encompasses a range of groups which collectively has been cited as being the largest and most disadvantaged ethnic minority group within Europe [1]. GRT is not a single homogenous group but is diverse with different beliefs, cultures and histories. Those defined as GRT include individuals who ethnically define themselves as GRT and those who are GRT through culture and occupation such as boaters or showmen [2]. These challenges in defining GRT populations makes it difficult to ascertain their population size within Europe which is believed to be over eight million [3].

GRT communities face stark inequalities that extend across the life course. It is estimated that as many as one-third of GRT individuals may be illiterate and many have never attended formal education [3]. A systematic review exploring the links between low literacy levels in GRT communities and health found that poor literacy contributed to increased psychological stress, disempowerment to self-advocate for their health needs, lower health literacy and challenges in navigating health systems to access care. Low literacy levels are further compounded by social exclusion, discrimination, poverty and poor living conditions.

The health inequalities experienced by GRT populations are well documented. They are more likely to develop a long-term health condition, experience infant and maternal mortality and to have a life expectancy of ten years less than the national average [4]. GRT communities face several barriers when accessing healthcare. For those who are mobile, frequent relocation makes sustained engagement with healthcare services challenging [5]. For settled GRT communities cited barriers include a pride in self-reliance, lack of understanding from healthcare professionals and a sense of shame for accessing health services for sensitive health needs [1].

There is a paucity of evidence relating to the oral health needs of GRT. Published data from several EU countries suggest that the inequalities GRT face extend to their dental needs [6–10]. A pilot study conducted in England found that 92% of GRT children involved had a moderate to high risk of developing future caries, which would have a significant adverse impact on their quality of life and further compound the inequalities they experience [11]. GRT groups have high levels of unmet need, some of the suggested determinants of poor oral health have been identified as; a lack of accessible and culturally appropriate information, distrust, anxiety about visiting the dentist and a historical absence of dental services reaching out and engaging with GRT communities [12]. One of the main consequences of this unequal access is the proclivity of GRTs to use emergency services as a substitute for routine care [13]. Outside this general trend, little is known about the inequities GRT populations face, including what impacts their access to care, and how best to address the oral health inequities they experience.

This review aims to map the evidence and identify knowledge gaps in oral health improvement and dental care access among GRT communities to direct future attempts to improve oral healthcare outcomes for these groups. There are four focused research questions that this review sought to answer:

1. *How do GRT communities across Europe utilise dental services, and to what extent can they access dental care?*
2. *What are the barriers and facilitators for GRT accessing dental services in Europe?*
3. *What oral health interventions and improvement strategies have been used in Europe to improve the oral health of GRT?*
4. *What factors affect the success of GRT oral health improvement in Europe?*

## Methods

Scoping reviews aim to map the breadth of literature on a topic and identify knowledge gaps, regardless of publication type; they are not limited to peer-reviewed studies [14]. This scoping review is particularly important as it synthesises both peer-reviewed and grey literature on an underserved population for which little is known about best practice. The review was conducted following the JBI methodology for scoping reviews [15], which uses the population, concept, and context of the issue under investigation as a framework to guide the development of research questions [15]. Each of these elements is detailed further below. The review protocol was not registered a priori.

### Inclusion and exclusion criteria

The search focused on oral health improvement and dental care access among GRT populations. The population of interest included both children and adults from this multi-ethnic group, which comprises a variety of subgroups who move freely across locations in Europe. Measures of access considered included registration, proximity to services, and patterns of service use. In terms of context, Europe was defined as the continent east of the Atlantic Ocean, north of the Mediterranean, and west of Asia [16]. The review drew upon both published and grey literature, including empirical studies, editorials, reports, surveys, and analyses. Searches were not restricted by publication date.

Items were included if they provided any level of evidence (including secondary evidence) or information from any source that contributed to answering the research question outlined above. The inclusion criteria are summarised below (Table 1):

**Table 1:** Inclusion and exclusion Criteria.

	Inclusion criteria	Exclusion criteria
Population	Any group that is part of the GRT Community, including children and adults	Any group not coming under the umbrella of the GRT community.
Concept	Studies that focus on oral health access or oral health improvement. All study types included.	Clinical and genetic studies
Context	Studies based in continental Europe and published in English. No date restrictions applied.	Studies conducted in countries outside Continental Europe or in other languages than English.

### Search strategy

A pilot search was undertaken on Scopus and Google to identify articles and reports. An experienced information specialist (LB) developed and conducted the searches on the 15^th^ of May, 2024. Relevant results were used to develop the search strategy for the review. Searches were undertaken on four databases, selected for their multidisciplinary breadth of coverage and dental subject focus: Embase (Ovid), Scopus (Elsevier), Web of Science (Clarivate) and Dentistry and Oral Sciences Source (EBSCOhost). The search strategy for the healthcare databases comprised two blocks of terms to represent the participants, including nomenclature used in European countries (Gypsy, Romany and Travellers) and the concept (health improvement and access). The search strategy for Embase Scopus, Web of Science and Dentistry & Oral Sciences Source (DOSS) is available in Appendix 1.

Grey literature was sought using targeted searches of the websites of relevant GRT charities, healthcare organisations, and professional bodies (see Appendix 2). Google searches were limited using the syntax and site-specific operators shown in Appendix 2, with screening restricted to the first 10 pages of results for each search. Empirical studies, secondary research, reports, opinion pieces, abstracts, editorials, and commentaries were eligible for inclusion. Following inclusion, papers were subject to reference list searching to identify additional relevant literature.

### Data management and selection process

Results were collated in EndNote for deduplication and in Rayyan for screening [17]. After duplicates were removed, two independent reviewers (JT-R and JB) screened items against pre-specified eligibility criteria. Potentially relevant items were then retrieved and collated in Rayyan for full-text independent screening by JT-R and RW. Any disagreements were resolved through consultation with a third reviewer (MP).

### Data extraction

Data were extracted by JT-R and verified by RW using a tool developed by the review team, which was refined following piloting with four articles. The extraction tool captured details about participants, concept, context, information sources, and all findings relevant to Research Questions 1-4. Extracted data were then subjected to thematic analysis to identify overarching themes within the body of literature [18].

### Data synthesis

Data synthesis was guided by the Arksey and O’Malley framework [19], which involves identifying research questions and relevant studies, selecting studies, tabulating data, and collecting and summarising results. The team (JT-R, MP, RW, JB, AM, JD) summarised and reviewed the results, enabling thematic analysis of the extracted content. Each document was coded inductively, with quotes and summaries extracted into an Excel sheet for analysis [20].

### Findings

**Fig. 1:**
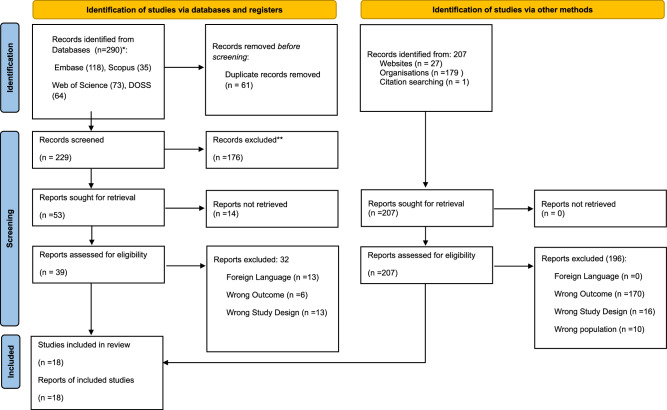
Prisma flow chart. Source: Page MJ, et al. BMJ 2021;372:n71. 10.1136/bmj.n71. PRISMA diagram of search and screening [49].

Figure 1 details the flow of information through the different phases of the scoping review.

A total of 497 records were identified from electronic databases, websites, organisations, and citation searching. After the removal of 61 duplicates, records were screened and assessed for eligibility. Eighteen studies met the inclusion criteria and were included in this scoping review, consisting of 7 peer-reviewed articles and 11 grey literature sources.

### Study characteristics

Study characteristics are given below and in Appendix 3. The studies included in the review involved one or more groups from within the Gypsy, Romany, and Traveller populations, with some focusing on only one of the groups rather than all three. The majority of papers put them together as a single population (56%), after which Travellers (11%) and Gypsies and Travellers (11%) were most commonly reported upon. Fifteen papers were in the United Kingdom (83%), one in the Republic of Ireland, one in Romania. One was from a European-wide data set. Methodological approaches varied widely. Mixed methods and qualitative work were the most commonly used methods.

Approaches to sampling varied. The most common was snowballing or recruitment through a mediator, e.g., using community nurses, dentists, dental nurses, Gypsy Liaison Officers and Health Visitors. A third of the papers did not describe any sampling method at all.

Age and gender were poorly reported. Most studies focused on adults, and only one focused on children and young adults aged 1–16 years [11]. Three others collected data on parents and children [21–23]. Only one reported on the participants’ sex [21], which was a gendered study involving only women and children. In all other cases, reporting on the gender split of participants was absent.

### Dental care access of GRT communities across Europe

In our analysis, we identified several patterns of access and attendance of GRT populations (RQ1).

#### Reliance on accident and emergency services

Multiple studies commented on the interaction of GRT populations with emergency services for oral health concerns. The 2021 Evidence for Equality National Survey stated that “*access to health and social care services in Britain was poorer for ‘Roma’ people compared with the White British group”* [24]. The lack of access to routine appointments and services means that there is a greater tendency for Gypsy, Romany or Traveller groups to rely on Accident and Emergency (A&E) services [25].

A study in Somerset found that “*approximately 60% of both Boaters and New Travellers would wish to utilise A&E for out-of-hours, preferring (at around 75%) to make use of walk-in clinics. Only a limited number of respondents (only one ‘ethnic’ Gypsy/Traveller) would consider going to an emergency dental service in an emergency, preferring even with dental issues to attend at A&E”* [26]. Deterioration of oral health to the point of pain or needing urgent dental services was a repeated theme, which we will discuss further in this paper.

#### Unauthorised sites experience worse access

A common theme emerged from the literature around unauthorised GRT sites, namely that “*those that travel and those that live on some types of unauthorised sites experience the worst access”* [27], when compared to those on authorised sites or regular housing. Nonetheless, housed GRT populations still have poorer access than the rest of the population (ibid). Mobile populations can experience increased distances to services (often without transport or poor public transport links), creating barriers to accessing routine appointments, thus increasing reliance upon A&E or, to a lesser extent, walk-in services [28].

#### Using mobile dental units

Several studies commented on mobile dental units (MDUs) usage [28, 29]; the findings were mainly positive regarding their ability to engage residents with oral health services. The studies also cover some of the disadvantages of this approach concerning the cost to patients and Local Authorities, e.g., “*the cost of an MDU can be prohibitive when considering whether or not to implement an outreach programme; both the initial start-up and ongoing costs can be high*” [11]. Other papers [29, 30] found similar concerns, as did the Bristol Traveller Project, which found running an MDU “*labour-intensive*” and dependent on having enough staff to provide treatment and drive a truck [31]. While MDUs can facilitate access, they also have some observable detracting factors. There were no data on whether they are a sustainable, long-term option for engagement and oral health improvement/preventative care.

### Barriers to oral health access and improvement

In this section, we first discuss the barriers noted in the literature, after which we cover facilitators in each area. A summary of the findings is given in Table 2 below.

**Table 2:** Summary table of barriers and facilitators to dental access and oral health improvement.

Barriers	Facilitators
Health literacy – booking appointments and reading medicine labels can be challenging. [23, 28, 35, 38, 43, 45]	
Cultural competence of the dental team – understanding of the culture of fear in the GRT population [43]. Awareness of GRT perceptions of hygiene [23, 33]	Community Health Workers - Can facilitate access and promote oral health for the GRT community and act as a trusted representative of health services. [21,22, 33, 35, 38]
Cost – accessing non-NHS services and health insurance is cost-prohibitive, reducing the likelihood of GRTs interacting with dental services. [27, 35,36]	
Physical mobility- Challenges monitoring unfixed population (feature in statistics), and reduced access to routine services. [23, 26, 44]	
Mobile Dental Units - Expensive to run and difficult to staff. [31]	Mobile Dental Units - Good engagement results, popular with communities and able to access unauthorised sites. Enables mobile populations to be accessed for oral health improvement work. [21, 28, 35, 48]

### Poor health literacy and limited understanding on how to access services

Literacy in general and health literacy, more specifically, are barriers to dental care for GRT populations. This includes “*knowing how to access and navigate health systems*” [1] and having the capacity to understand health information [24]. This was part of a broader communications issue regarding the use of technology in practices, such as touch screens [21].

### Structural barriers to access

Accessing and documenting the opinions of GRT populations can be challenging [32], partly due to the paucity of granular data recording by minoritised ethnic group [12]. Consequently, studies found it hard to associate clinical staff records with specific groups, as the GRT community was *“absorbed in their caseload”* [ibid].

#### Fear and cultural factors

GRT populations were described as avoiding treatment *“due to fear or lack of perceived benefit*” [33]. The Welsh Government [34] reported that GRT groups were not found to resist mainstream health services due to lack of choice but rather cultural factors.

A study of GRT, boater and showman populations in Somerset, UK found that “*47% of respondents see their dentist at least annually*” [26]. They matched the population’s self-assessment of dental treatment need against a clinician’s opinion. They found 80% agreement in cases, suggesting that a lack of understanding did not explain the lack of access.

Fear, however, can span multiple family or group members; *“transgenerational beliefs within the community are strong. Fear and experiences of previous generations still resonate and inhibit current patients from taking advantage of treatments offered to them”* [33]. Consequently, access patterns can manifest for long periods and be challenging to alter.

Differences between service providers and GRT cultures inhibit the development of trusting relationships and, therefore, diminish GRT access to oral health care. There is “*a lack of cultural competence and knowledge on the part of healthcare providers”* [21]. To address these issues, dental teams are recommended to be *“aware of the cultural and environmental factors affecting dental health and service use”* [23], such as literacy levels.

#### Costs prohibit access to dental care

Four papers considered the costs associated with dental access, *“which resulted in years of neglecting their oral health”* [35]. Not being able to see a dentist resulted in reduced well-being, pain and worsening symptoms.

In Europe, health insurance policies are more common than in the UK as a means to pay for treatment. In Romania, “*Dental care was commonly needed, but dental services are not covered within health insurance policies, and costs are too high for most*” [36]. The article suggested that bribes were sometimes required either for access to treatment, or impacted the standard of treatment offered and the attitude of healthcare professionals.

The implications are that bribes appear to be perceived as necessary to access care that is not included in health insurance (such as dentistry). No studies considered cost as a barrier for health improvement interventions.

#### Physical mobility of GRTs affects access

Travellers who are mobile experience additional unique barriers. Travellers on transit or unauthorised sites reported the most difficulties, *“Many Travellers on these sites said that they had not had a problem getting to a dentist because they had never tried*” [23]. The most common barrier was being moved on. Therefore, waiting for appointments made accessing routine care difficult. Mobility also caused a reluctance to register with a dental practice. Further, access was a gendered issue; “*transport was an added barrier for many women”* [23]. This was because they were also the most isolated group (due to gendered differences in work) on transit or unauthorised sites, often many miles from the nearest town.

### Facilitators for oral health access and improvement

Regarding oral health improvement, three studies provided brief insights into facilitators. Two papers pointed to community-facing healthcare roles to promote services.

#### Community Health Workers help foster trust in dental care services

Community-based liaison roles were commonly the bridge between GRT populations and health services. For example, by supporting the registration of patients without proof of address or facilitating oral health promotion projects [36]. Using home visits, a London-based study found that “*collaborating with a familiar community nurse also helped to allay some of the Travellers’ reservations and uncertainties associated with attending the MDU”* [11].

In 2007, the Irish Government Health Service Executive used community health workers for health improvement, whose work was noted as a facilitator in the programme. A systematic review [22] also highlighted a study of the Romany population in the Czech Republic [37] using health and social care assistants whose role included educating and motivating community members around healthcare. This had positive outcomes, including increased Roma attending preventative dental examinations.

Regular interaction and communication with community-facing healthcare staff generated trust between GRT and health professionals and their services [38]. These roles promote flexibility in the system for GRT residents who lack addresses or formal documentation or need flexible drop-in appointments rather than pre-booking timeslots [33]. Working with trusted organisations or community-based partners would require feasible flexible commissioning approaches [27].

#### Mobile Dental Units

Mobile Dental Units (MDUs) support oral health access and improvement. It was found that “*verbal feedback from families, after engagement with the MDU and [Special Care Dentistry] team, was positive and encouraging*” [11], illustrating that participants appreciated the mobile service. A similar service was previously set up by the Bristol Traveller Health Project [29].

#### Oral health interventions and improvement strategies

No peer-reviewed literature on GRT oral health improvement was found. The grey literature search identified one Scotland-wide programme (Childsmile) which distributes dental packs and provides supervised tooth-brushing in primary schools in disadvantaged populations (including GRT) and all nurseries [21]. This universal programme aims to reduce inequalities in oral health and access to dental services rather than being specifically for or targeted at GRTs. Another study also noted that health visiting teams who are resourced to have oral health packs and deliver oral health promotion can facilitate the oral health education of whole families whilst supporting dental access for individuals. [12], i.e., any access project should be part of an improvement exercise.

### Ethics declaration

This study used publicly available information and therefore ethical approval was not required.

## Discussion

### Summary of findings

This scoping review identified a limited evidence base on dental services access and oral health improvement among GRT populations in Europe. The literature is dominated by studies describing barriers to access, with far fewer evaluations of oral health improvement interventions. Key barriers included low health literacy, structural and administrative constraints, cost, mobility-related disruption to continuity of care, and limited cultural competence within dental services. Facilitators were less frequently reported but included community health worker models, mobile dental services, and trusted relationships with providers. Overall, the findings suggest that oral health inequalities in GRT populations are driven predominantly by structural and system-level determinants rather than individual behaviours, with trust emerging as a central mechanism influencing engagement.

### Comparison with existing literature and recommendations

#### Oral health inequalities in a broader health context

The oral health inequalities identified in this review reflect wider patterns of health disadvantage experienced by GRT communities across Europe. Similar barriers are observed in maternity care, chronic disease management, vaccination uptake, and mental health services, where GRT populations experience reduced preventive care engagement and greater reliance on emergency services [4, 5, 39, 40]. The use of accident and emergency services for dental problems, therefore, aligns with a broader pattern of fragmented healthcare access rather than a condition-specific issue [5].

These parallels suggest that oral health inequalities should be understood within the wider context of social exclusion, discrimination, and system inflexibility affecting multiple health domains [1, 39, 41, 42]. However, comparative research across health outcomes remains limited, restricting a more integrated understanding of GRT health inequalities.

#### Access, service design, and system limitations

Findings highlight that current dental service models are often poorly aligned with the needs of GRT populations. Structural barriers such as registration requirements, proof of address, and inflexible appointment systems are consistently reported across healthcare settings and reflect broader patterns of restricted healthcare access experienced by GRT communities particularly those who are mobile or living on unauthorised sites [1, 5, 23].

Mobile dental units and outreach services can improve access, particularly for populations on unauthorised sites, but evidence of sustained oral health improvement is limited. In addition, these models are resource-intensive, and there is insufficient evidence on long-term sustainability or comparative effectiveness across different GRT subgroups.

#### Communication, literacy, and cultural competence

Health literacy was consistently identified as a barrier to accessing dental care, particularly in relation to navigating systems and understanding preventive care. However, wider GRT health literature emphasises that literacy-related barriers are closely linked to structural complexity, digital exclusion, and service design rather than individual capacity alone [39, 41].

Cultural competence among dental teams was frequently cited as important but remains poorly defined and inconsistently evaluated. While communication strategies such as verbal engagement and flexible appointment systems are recommended, there is limited evidence on their effectiveness or scalability [1, 5]. This represents a key gap in understanding how to operationalise culturally responsive care in routine dental services.

#### Trust as a central determinant of engagement

Trust emerged as a key cross-cutting factor influencing service uptake and continuity of care. It is shaped by previous experiences of discrimination, perceived judgement, and continuity of relationships with providers [39]. This reflects broader GRT health literature, where mistrust of statutory services is linked to historical exclusion and negative healthcare encounters [4, 5]. Community health worker models appear to support trust-building by acting as intermediaries between services and GRT communities [1, 12]. The co-production of services and the involvement of community-based oral health roles may help build trust within GRT communities [30].

However, the evidence base remains largely descriptive, with limited evaluation of how trust develops or translates into improved oral health outcomes. Future research should conceptualise trust as a dynamic process shaped by both interpersonal and structural factors.

#### Interventions and evidence gaps

There is a notable lack of peer-reviewed studies evaluating oral health improvement interventions for GRT populations. Existing initiatives are predominantly embedded within broader deprivation-focused programmes rather than specifically targeting GRT communities. As a result, there is limited evidence on effectiveness in improving oral health outcomes.

In addition, there is an absence of economic evaluation of outreach models, mobile services, and community-based interventions. This limits the ability to assess value for money and sustainability, contributing to continued reliance on short-term or pilot interventions [5].

#### Unanswered questions and future research

Key gaps remain in intervention research, particularly studies evaluating oral health outcomes rather than service utilisation. Longitudinal and comparative studies are needed to assess the effectiveness of different service delivery models, including mobile and community-based care.

There is also a need for research that disaggregates outcomes across different GRT subgroups, as treating GRT populations as homogeneous may obscure important differences in access and need [39]. Methodological innovation, including embedded and community-led research approaches, may help address challenges associated with mobility and follow-up.

Additionally, it is recommended that the UK health services’ approach to ethnic monitoring be revised to more accurately capture the numbers and locations of GRTs accessing dental care [35]. This monitoring could be applied across A&E, Emergency Dental Services, Dental Access Centres, and Special Care Dentistry Services [35].

Finally, future research should adopt a more integrated public health perspective, examining oral health alongside other health outcomes to better understand shared structural determinants of inequality.

### Limitations

This scoping review has several limitations relating to the included evidence base. Many studies had small sample sizes and were conducted in limited settings, such as children’s centres or authorised sites. Recruitment was often opportunistic and unstructured, which reduces the generalisability and transferability of findings and may result in underrepresentation of segments of the GRT population.

For quantitative studies, data quality was a key limitation, particularly inaccurate or inconsistent ethnic categorisation of GRT populations in healthcare datasets. Aggregated categories such as “other” can obscure important differences within GRT communities [25], and missing data remain common, particularly for individuals not registered with services or outside mainstream education.

In addition, relevant evidence may exist in non-English languages but was not included due to translation constraints, introducing potential language bias. Eighteen non-English papers were excluded, equivalent to the number of included studies, suggesting that a substantial body of evidence may not be represented in this review.

## Conclusions

GRT communities experience significantly reduced access to routine dental care and greater reliance on emergency services due to structural, financial, and mobility-related barriers. Key barriers include low health literacy, service inflexibility, cost, and limited cultural competence, while facilitators are mainly community health workers, outreach models, and trust-building approaches. Evidence on oral health improvement interventions remains extremely limited, with few evaluated or GRT-specific programmes identified. Across the literature, trust, service accessibility, and culturally responsive care appear to be the main determinants of successful engagement. Overall, the evidence base remains focused on describing inequities rather than testing solutions, highlighting the need for robust, co-produced intervention research.

## Data Availability

The datasets generated and/or analysed during the current study are publicly available.

## Appendix

**Appendix 1**: Search Terms and Strategy

**Embase** < **1974 to 2024 May 15** > 

**15/05/24**

1. “romani (people)”/906
2. ((gypsy$1 or gypsies or gipsy$1 or gipsies) not (moth or moths or gypsyty3 or gypsy ty3 or ty3gypsy or ty3 gypsy)).ti,ab,kf.2045
3. (roma or romas or romany$1 or romani or romanis or romanies).ti,ab,kf.3403
4. (arli or arlis or ashkali or ashkalis or aurari or auraris or balkan egyptian or balkan egyptians or bashalde or bashaldes or boyash$1 or churari or churaris or cigano or ciganos or erlide or erlides or gitano or gitanos or gitans or horahane or horahanes or kalderash$1 or lalleri or lalleris or lingurari or linguraris or lovari or lovaris or ludar or ludars or ludari or ludaris or luri or luris or machvaya or machvayas or manouche or manouches or manush or manushs or manushes or modgar or modgars or modyar or modyars or romanichal or romanichals or romanichel or romanichels or romanis?l or romanis?ls or romungro or romungros or rudari or rudaris or tsigane or tsiganes or ungaritza or ungaritzas or ursari or ursaris or yerlii or yerliis or zl?tari or zl?taris).ti,ab,kf.80
5. (sinti or sinta or sinte or sintis or sintas or sintes or ceardannan$1 or yenish$1 or yeniche$1 or jenische$1 or quinqui$1 or mercheros$1).ti,ab,kf.79
6. kale.ti,ab,kf. not (exp Brassica/ or brassica$.ti,ab,kf.)318
7. (fairground$1 or fair-ground$1 or funfair$1 or fun-fair$1 or showmen$1 or show-men$1 or showwomen$1 or show-women$1 or showperson$1 or show-person$1 or showpeople$1 or show-people$1 or show communit$ or show travel?er$1 or forains industriel).ti,ab,kf.293
8. (circuses or circus men$1 or circus women$1 or circus person$1 or circus people$1 or circus communit$ or circus travel?er$1).ti,ab,kf. 62
9. (bargee$1 or canal boat$1 or barge$1 or boat-dwell$).ti,ab,kf.326
10. (pavee$1 or minceir$ or lucht$1 or luchd$1 or itinerants).ti,ab,kf.32
11. (travel?er$1 and (communit$ or family or families or irish or ireland$ or eire or wales or welsh or scottish or scotland$1 or highland$1 or norwegian$1 or norway$1 or newage or new-age or itinerant$1 or minorit$ or ethnic$ or halting site$1 or caravan$1)).ti,ab,kf.2065
12. (travel?er$1 adj1 (people$ or person or persons or children$1 or child$1 or men or mens or male$1 or women$1 or female$1 or population$1 or group$1 or site or sites)).ti,ab,kf. 450
13. occupational travel?er$1.ti,ab,kf. 9
14. (travel?ing adj5 (communit$ or family or families or irish or ireland$ or eire or wales or welsh or scottish or scotland$1 or highland$1 or norwegian$1 or norway$1 or newage or new-age or itinerant$1 or minorit$ or ethnic$ or site$1 or caravan$1)).ti,ab,kf. 554
15. (travel?er$1 or travel?ing).ti,ab,kf. and (migrant/ or vulnerable population/ or minority group/ or minority health/ or ethnic group/) 499
16. travel?er$1.ti. or travel?er$1.ab. /freq=2 10297
17. exp Travel/ or Travel Medicine/ or travel related disease/ or travel/ or travel restriction/ or emporiatics/ or “travel medicine & infectious disease”.jn. or (travel or (travel$ adj3 (oversea$ or abroad$ or international$ or vacation$ or holiday$)) or (return$ adj1 travel?er$)).ti,ab,kf. 100794
18. 16 not 17 1748
19. or/1-15,18 10904
20. dental health/ 5447
21. dental procedure/35014
22. exp tooth disease/249689
23. exp dentist/30262
24. (oral adj3 (health* or hygiene or care)).ab,kw,ti. 60679
25. dental.ab,kw,ti. 260512
26. ((tooth adj3 (health* or hygiene or care or brush* or floss*)) or toothbrush*).ab,kw,ti.9806
27. (teeth adj3 (health* or hygiene or care or brush* or floss*)).ab,kw,ti. 5225
28. dentist*.ab,kw,ti. 86061
29. or/20-28 504544
30. 19 and 29 118

**Scopus**:

**15/05/24**

(((TITLE-ABS-KEY (gypsy* OR gypsies OR gipsy* OR gipsies OR roma OR romas OR romany* OR romani OR romanies OR romanis) OR TITLE-ABS-KEY (arli OR arlis OR ashkali OR ashkalis OR aurari OR auraris OR “balkan egyptian*“ OR bashalde OR bashaldes OR boyash* OR churari OR churaris OR cigano OR ciganos OR erlide OR erlides OR gitano OR gitanos OR gitans OR horahane OR horahanes OR kalderash* OR lalleri OR lalleris OR lingurari OR linguraris OR lovari OR lovaris OR ludar OR ludars OR ludari OR ludaris OR luri OR luris OR machvaya OR machvayas OR manouche OR manouches OR manush OR manushs OR manushes OR modgar OR modgars OR modyar OR modyars OR romanichal OR romanichals OR romanichel OR romanichels OR romanis?l OR romanis?ls OR romungro OR romungros OR rudari OR rudaris OR tsigane OR tsiganes OR ungaritza OR ungaritzas OR ursari OR ursaris OR yerlii OR yerliis OR zl?tari OR zl?taris) OR TITLE-ABS-KEY (sinti OR sinta OR sinte OR sintis OR sintas OR sintes OR ceardannan* OR yenish* OR yeniche* OR jenische* OR quinqui* OR mercheros*) OR TITLE-ABS-KEY (fairground* OR fair-ground* OR funfair* OR fun-fair* OR showmen* OR show-men* OR showwomen* OR show-women* OR showperson* OR show-person* OR showpeople* OR show-people* OR show-communit* OR show-travel?er* OR “forains industriel”) OR TITLE-ABS-KEY (circuses OR circus-men* OR circus-women* OR circus-person* OR circus-people* OR circus-communit* OR circus-travel?er*))) OR ((TITLE-ABS-KEY (bargee* OR canal-boat* OR barge* OR boat-dwell*) OR TITLE-ABS-KEY (pavee* OR minceir* OR lucht* OR luchd* OR itinerants) OR TITLE-ABS-KEY (travel?er* AND (communit* OR family OR families OR irish OR ireland* OR eire OR wales OR welsh OR scottish OR scotland* OR highland* OR norwegian* OR norway* OR newage OR new-age OR itinerant* OR minorit* OR ethnic* OR halting-site* OR caravan*)) OR TITLE-ABS-KEY (travel?er* W/1 (people* OR person OR persons OR children* OR child* OR men OR mens OR male* OR women* OR female* OR population* OR group* OR site OR sites)) OR TITLE-ABS-KEY (circuses OR circus-men* OR circus-women* OR circus-person* OR circus-people* OR circus-communit* OR circus-travel?er*) OR TITLE-ABS-KEY ((occupational-travel?er*))) OR (TITLE-ABS-KEY (travel?ing W/5 (communit* OR family OR families OR irish OR ireland* OR eire OR wales OR welsh OR scottish OR scotland* OR highland* OR norwegian* OR norway* OR newage OR new-age OR itinerant* OR minorit* OR ethnic* OR site* OR caravan*))) OR ((TITLE-ABS-KEY (travel?ing OR travel?er*) AND KEY (migrant OR vulnerable OR ethnic OR minorities OR minority))))

AND

((TITLE-ABS-KEY (dental OR dentist*) AND TITLE-ABS-KEY (((oral) W/3 (health* OR hygiene OR care))) OR TITLE-ABS-KEY (((tooth OR teeth) W/3 (health* OR hygiene OR care OR brush* OR floss*)) OR toothbrush*)))

**Web of Science**

**15/05/24**

TS = (gypsy* OR gypsies OR gipsy* OR gipsies OR roma OR romas OR romany* OR romani OR romanies OR romanis OR arli OR arlis OR ashkali OR ashkalis OR aurari OR auraris OR “balkan egyptian*“ OR bashalde OR bashaldes OR boyash* OR churari OR churaris OR cigano OR ciganos OR erlide OR erlides OR gitano OR gitanos OR gitans OR horahane OR horahanes OR kalderash* OR lalleri OR lalleris OR lingurari OR linguraris OR lovari OR lovaris OR ludar OR ludars OR ludari OR ludaris OR luri OR luris OR machvaya OR machvayas OR manouche OR manouches OR manush OR manushs OR manushes OR modgar OR modgars OR modyar OR modyars OR romanichal OR romanichals OR romanichel OR romanichels OR romanis?l OR romanis?ls OR romungro OR romungros OR rudari OR rudaris OR tsigane OR tsiganes OR ungaritza OR ungaritzas OR ursari OR ursaris OR yerlii OR yerliis OR zl?tari OR zl?taris OR sinti OR sinta OR sinte OR sintis OR sintas OR sintes OR ceardannan* OR yenish* OR yeniche* OR jenische* OR quinqui* OR mercheros* OR fairground* OR fair-ground* OR funfair* OR fun-fair* OR showmen* OR show-men* OR showwomen* OR show-women* OR showperson* OR show-person* OR showpeople* OR show-people* OR show-communit* OR show-travel?er* OR “forains industriel” OR circuses OR circus-men* OR circus-women* OR circus-person* OR circus-people* OR circus-communit* OR circus-travel?er* OR bargee* OR canal-boat* OR barge* OR boat-dwell* OR pavee* OR minceir* OR lucht* OR luchd* OR itinerants OR ((travel?er* OR travel?ing) NEAR/5 (communit* OR family OR families OR irish OR ireland* OR eire OR wales OR welsh OR scottish OR scotland* OR highland* OR norwegian* OR norway* OR newage OR new-age OR itinerant* OR minorit* OR ethnic* OR halting-site* OR caravan*)) OR (travel?er* NEAR/1 (people* OR person OR persons OR children* OR child* OR men OR mens OR male* OR women* OR female* OR population* OR group* OR site OR sites)) OR circuses OR circus-men* OR circus-women* OR circus-person* OR circus-people* OR circus-communit* OR circus-travel?er* OR occupational-travel?er* OR ((travel?ing OR travel?er*) AND (migrant OR vulnerable OR ethnic OR minorities OR minority)))

AND

TS = (dental OR dentist OR ((oral) NEAR/3 (health* OR hygiene OR care)) OR ((tooth OR teeth) NEAR/3 (health* OR hygiene OR care OR brush* OR floss*)) OR toothbrush*)

**DOSS**

**15/05/24**

TI (gypsy* OR gypsies OR gipsy* OR gipsies OR roma OR romas OR romany* OR romani OR romanies OR romanis OR arli OR arlis OR ashkali OR ashkalis OR aurari OR auraris OR “balkan egyptian*“ OR bashalde OR bashaldes OR boyash* OR churari OR churaris OR cigano OR ciganos OR erlide OR erlides OR gitano OR gitanos OR gitans OR horahane OR horahanes OR kalderash* OR lalleri OR lalleris OR lingurari OR linguraris OR lovari OR lovaris OR ludar OR ludars OR ludari OR ludaris OR luri OR luris OR machvaya OR machvayas OR manouche OR manouches OR manush OR manushs OR manushes OR modgar OR modgars OR modyar OR modyars OR romanichal OR romanichals OR romanichel OR romanichels OR romanis?l OR romanis?ls OR romungro OR romungros OR rudari OR rudaris OR tsigane OR tsiganes OR ungaritza OR ungaritzas OR ursari OR ursaris OR yerlii OR yerliis OR zl?tari OR zl?taris OR sinti OR sinta OR sinte OR sintis OR sintas OR sintes OR ceardannan* OR yenish* OR yeniche* OR jenische* OR quinqui* OR mercheros* OR fairground* OR fair-ground* OR funfair* OR fun-fair* OR showmen* OR show-men* OR showwomen* OR show-women* OR showperson* OR show-person* OR showpeople* OR show-people* OR show-communit* OR show-travel?er* OR “forains industriel” OR circuses OR circus-men* OR circus-women* OR circus-person* OR circus-people* OR circus-communit* OR circus-travel?er* OR bargee* OR canal-boat* OR barge* OR boat-dwell* OR pavee* OR minceir* OR lucht* OR luchd* OR itinerants OR ((travel?er* OR travel?ing) N5 (communit* OR family OR families OR irish OR ireland* OR eire OR wales OR welsh OR scottish OR scotland* OR highland* OR norwegian* OR norway* OR newage OR new-age OR itinerant* OR minorit* OR ethnic* OR halting-site* OR caravan*)) OR (travel?er* N1 (people* OR person OR persons OR children* OR child* OR men OR mens OR male* OR women* OR female* OR population* OR group* OR site OR sites)) OR circuses OR circus-men* OR circus-women* OR circus-person* OR circus-people* OR circus-communit* OR circus-travel?er* OR occupational-travel?er* OR ((travel?ing OR travel?er*) AND (migrant OR vulnerable OR ethnic OR minorities OR minority))) OR AB (gypsy* OR gypsies OR gipsy* OR gipsies OR roma OR romas OR romany* OR romani OR romanies OR romanis OR arli OR arlis OR ashkali OR ashkalis OR aurari OR auraris OR “balkan egyptian*“ OR bashalde OR bashaldes OR boyash* OR churari OR churaris OR cigano OR ciganos OR erlide OR erlides OR gitano OR gitanos OR gitans OR horahane OR horahanes OR kalderash* OR lalleri OR lalleris OR lingurari OR linguraris OR lovari OR lovaris OR ludar OR ludars OR ludari OR ludaris OR luri OR luris OR machvaya OR machvayas OR manouche OR manouches OR manush OR manushs OR manushes OR modgar OR modgars OR modyar OR modyars OR romanichal OR romanichals OR romanichel OR romanichels OR romanis?l OR romanis?ls OR romungro OR romungros OR rudari OR rudaris OR tsigane OR tsiganes OR ungaritza OR ungaritzas OR ursari OR ursaris OR yerlii OR yerliis OR zl?tari OR zl?taris OR sinti OR sinta OR sinte OR sintis OR sintas OR sintes OR ceardannan* OR yenish* OR yeniche* OR jenische* OR quinqui* OR mercheros* OR fairground* OR fair-ground* OR funfair* OR fun-fair* OR showmen* OR show-men* OR showwomen* OR show-women* OR showperson* OR show-person* OR showpeople* OR show-people* OR show-communit* OR show-travel?er* OR “forains industriel” OR circuses OR circus-men* OR circus-women* OR circus-person* OR circus-people* OR circus-communit* OR circus-travel?er* OR bargee* OR canal-boat* OR barge* OR boat-dwell* OR pavee* OR minceir* OR lucht* OR luchd* OR itinerants OR ((travel?er* OR travel?ing) N5 (communit* OR family OR families OR irish OR ireland* OR eire OR wales OR welsh OR scottish OR scotland* OR highland* OR norwegian* OR norway* OR newage OR new-age OR itinerant* OR minorit* OR ethnic* OR halting-site* OR caravan*)) OR (travel?er* N1 (people* OR person OR persons OR children* OR child* OR men OR mens OR male* OR women* OR female* OR population* OR group* OR site OR sites)) OR circuses OR circus-men* OR circus-women* OR circus-person* OR circus-people* OR circus-communit* OR circus-travel?er* OR occupational-travel?er* OR ((travel?ing OR travel?er*) AND (migrant OR vulnerable OR ethnic OR minorities OR minority))) OR SU (gypsy* OR gypsies OR gipsy* OR gipsies OR roma OR romas OR romany* OR romani OR romanies OR romanis OR arli OR arlis OR ashkali OR ashkalis OR aurari OR auraris OR “balkan egyptian*“ OR bashalde OR bashaldes OR boyash* OR churari OR churaris OR cigano OR ciganos OR erlide OR erlides OR gitano OR gitanos OR gitans OR horahane OR horahanes OR kalderash* OR lalleri OR lalleris OR lingurari OR linguraris OR lovari OR lovaris OR ludar OR ludars OR ludari OR ludaris OR luri OR luris OR machvaya OR machvayas OR manouche OR manouches OR manush OR manushs OR manushes OR modgar OR modgars OR modyar OR modyars OR romanichal OR romanichals OR romanichel OR romanichels OR romanis?l OR romanis?ls OR romungro OR romungros OR rudari OR rudaris OR tsigane OR tsiganes OR ungaritza OR ungaritzas OR ursari OR ursaris OR yerlii OR yerliis OR zl?tari OR zl?taris OR sinti OR sinta OR sinte OR sintis OR sintas OR sintes OR ceardannan* OR yenish* OR yeniche* OR jenische* OR quinqui* OR mercheros* OR fairground* OR fair-ground* OR funfair* OR fun-fair* OR showmen* OR show-men* OR showwomen* OR show-women* OR showperson* OR show-person* OR showpeople* OR show-people* OR show-communit* OR show-travel?er* OR “forains industriel” OR circuses OR circus-men* OR circus-women* OR circus-person* OR circus-people* OR circus-communit* OR circus-travel?er* OR bargee* OR canal-boat* OR barge* OR boat-dwell* OR pavee* OR minceir* OR lucht* OR luchd* OR itinerants OR ((travel?er* OR travel?ing) N5 (communit* OR family OR families OR irish OR ireland* OR eire OR wales OR welsh OR scottish OR scotland* OR highland* OR norwegian* OR norway* OR newage OR new-age OR itinerant* OR minorit* OR ethnic* OR halting-site* OR caravan*)) OR (travel?er* N1 (people* OR person OR persons OR children* OR child* OR men OR mens OR male* OR women* OR female* OR population* OR group* OR site OR sites)) OR circuses OR circus-men* OR circus-women* OR circus-person* OR circus-people* OR circus-communit* OR circus-travel?er* OR occupational-travel?er* OR ((travel?ing OR travel?er*) AND (migrant OR vulnerable OR ethnic OR minorities OR minority)))

**Appendix 2:** Grey Literature search strategy

Database	Search string
Google [27] - Searches limited to the first 10 pages of Google results.	(“gypsy” OR “ Romani” OR “traveller”) (report OR Evaluation) (“oral” or “Dental”) (“gypsy” OR “ Romani” OR “traveller”) (report OR Evaluation) (“oral” or “Dental” (site:nhs.uk OR site:gov.uk OR site:ac.uk) (“gypsy” OR “ Romani” OR “traveller”) (report OR Evaluation) (site:nhs.uk OR site:gov.uk OR site:ac.uk) (barriers OR enablers OR facilitators)
Kings Fund [1]	(Dental) AND (Gypsy OR Roma OR Traveller) (Oral) AND Gypsy OR Roma OR Traveller)
NICE Evidence [9]	(Dental) AND (Gypsy OR Roma OR Traveller) [9] (Oral) AND Gypsy OR Roma OR Traveller)
Open Grey [17]	(gypsy OR Romani OR traveller OR Oral OR Dental) AND (UK) [16] (gypsy OR Romani OR traveller OR Oral OR Dental) AND (Ireland) [1]
Health Foundation [4]	(Gypsy OR Roma OR Traveller) [4]
Healthwatch [0]	(Gypsy OR Roma OR Traveller) [0]
British Dental Association [0]	(Gypsy OR Roma OR Traveller) [0]
Health Education England [0]	(Gypsy OR Roma OR Traveller) [0]
NHS Education for Scotland [0]	(Gypsy OR Roma OR Traveller) [0]
Health and Social Care Committee [0]	(Gypsy OR Roma OR Traveller) [0]
College of General Dentistry [0]	(Gypsy OR Roma OR Traveller) [0]
Faculty of Dental Surgery of the Royal College of Physicians and Surgeons of Glasgow [0]	(Gypsy OR Roma OR Traveller) [0]
Faculty of Dental Surgery of the Royal College of Surgeons of Edinburgh [100]	(Gypsy OR Roma OR Traveller) [100]
Dental School Council [0]	(Gypsy OR Roma OR Traveller) [0]
Health Education and Improvement Wales [0]	(Gypsy OR Roma OR Traveller) [0]
Association of Dental Groups [1]	(Gypsy OR Roma OR Traveller) [1]
Northern Ireland Government [6]	(Dental) AND (Gypsy OR Roma OR Traveller) [6]
Welsh Government [6]	(Dental) AND (Gypsy OR Roma OR Traveller) [6]
Scottish Ireland Government [0]	(Dental) AND (Gypsy OR Roma OR Traveller) [0]
Faculty of Dental Surgery of the Royal College of Surgeons of England [0]	(Dental) AND (Gypsy OR Roma OR Traveller) [0]
Friends Families and Travellers [43]	Dent* [44] Oral [7]
Local Dental Committees [0]	(Gypsy OR Roma OR Traveller) [0]

**Appendix 3:** Study characteristics

Study	Type	Sampling	Setting	Population	Sample size
[11] Doughty et al., (2016)	Pilot evaluation	Opportunistic sampling using community nurses, dentists and dental nurses.	Traveller site in Hackney, London, England	Travellers (1- 16 years, gender not reported)	37
[45] Dyer et al., (2023)	Rapid Review	Evidence synthesised of recent, UK-based research	Any UK dental or oral health setting	GRT	31 studies included (1 GRT study)
[23] Edwards and Watt (1997)	Mixed methods (interviews and survey)	Gypsy Liaison Officers and Health Visitors were used to make introductions to participants.	Inside properties of Traveller sites in East Hertfordshire, England	English Gypsies and Irish Travellers	43 (interview) 72 (survey and clinical screening)
[36] George et al., (2018)	Qualitative study	Convenience sampling through parent’s meetings at a Children’s Community Centre	Romania	Romany only	18 Interviews (11 family members and 7 staff members)
[21] McFadden et al., (2018)	Systematic review	21 Databases and a Grey Lit search	32 countries (31 European countries and Canada).	GRT	99 studies included
[38] Siebelt et al., (2017)	Abstract	Not stated	Scotland & England	GRT	213 interviews (174 GRT, 39 service providers) 76 in co-production workshops
[33] Walshaw and Ireland, (2017)	Clinical Case Study	Not stated	England	GRT in Oxfordshire	Not stated
[46] The Association of Dental Groups (2019)	Select Committee Evidence	Not stated	Not stated	Gypsy and Irish Traveller	Not stated
[22] Irish Government Health Service Executive (2007)	Quantitative evaluation	Details drawn from the Traveller register.	County Offaly Republic of Ireland	Travellers	Mothers (n = 89) (with children under 16 years of age) Adults (n = 212) (inclusive of mothers n = 89).
[12] Mathews et al., (2010)	Qualitative study (ethnographic)	Opportunistic and structured. The structured work was conducted in Children and Family Centres	England	GRT in Sussex	Not stated
[1] McFadden et al., (2018)	Mixed methods: Literature review (realist), surveys and qualitative evaluation	Access to participants via third-sector organisations	UK	Romany Gypsy, Irish Traveller and Eastern European Romany communities	37 interviews, 196 survey responses, 26 studies were included in the review
[47] Power, C (2004)	Qualitative study	Not stated	England	Irish Travellers	Not stated
[27] Welsh Government (2015)	White paper	Not stated	Wales	GRT	Not stated
[25] A Bolder Healthier Birmingham (2023)	Mixed methods: literature reviews and analysis of routine data sets (NHS)	Not stated	UK	GRT	Not stated
[26] Greenfields and Lowe (2013)	Mixed methods - survey and interviews	Recruitment was part of Gypsy Traveller Accommodation Assessments (GTAAs)	England	Gypsy, Traveller, Boater, Showman and Romany in Somerset	66 interviews, 40 health professionals (surveyed)
[35] McFadden et al., (2023)	Qualitative study	Community Health Workers recruited participants are part of their routine work at registered GRT sites.	Scotland	GRT	5 Focus groups, 3 informal visits 1 workshop, 15 interviews
[34] Welsh Government (2003)	Policy Review	Data drawn from a mixture of authorised and unauthorised sites	Wales	GRT	Not Stated
[44] NHS England & Improvement (2021)	White Paper	Not stated	England	GRT	Not stated
